# Modal Analysis of the Human Brain Using Dynamic Mode Decomposition

**DOI:** 10.3390/bioengineering11060604

**Published:** 2024-06-12

**Authors:** Jayse McLean, Mehran Fereydoonpour, Mariusz Ziejewski, Ghodrat Karami

**Affiliations:** Department of Mechanical Engineering, North Dakota State University, Fargo, ND 58102, USA; jayse.mclean.1@ndsu.edu (J.M.); mehran.fereydoonpour@ndsu.edu (M.F.); mariuz.ziejewski@ndsu.edu (M.Z.)

**Keywords:** brain injury, modal analysis, dynamic mode decomposition

## Abstract

The majority of observations and criteria related to brain injuries predominantly focus on acceleration and forces, leaving the understanding of the brain in the frequency domain relatively limited. The impact of an injury can be more profound when considering the brain’s resonant frequencies in conjunction with external applied loading and motion. This paper employs a finite element method to conduct an analysis of a human brain under impacts from various angles on the human head. A numerical technique, specifically dynamic mode decomposition (DMD), is utilized to extract modal properties for brain tissue in regions proximate to the corpus callosum and brain stem. Three distinct modal frequencies have been identified, spanning the ranges of 44–68 Hz, 68–155 Hz, and 114–299 Hz. The findings underscore the significance of impact angle, displacement direction, and the specific region of the brain in influencing the modal response of brain tissue during an impact event.

## 1. Introduction

The human brain, despite its immense power and complexity, is remarkably fragile and susceptible to injury. Traumatic brain injury (TBI) resulting from external forces introduces alterations to brain function. Mild traumatic brain injuries (MTBIs), commonly known as concussions, have become an epidemic in American sports, notably in football. Impact on the human brain initiates waves of vibration that propagate and attenuate at varying rates, influenced by the magnitude and direction of loading. Similar to any dynamic system, the human brain exhibits natural frequencies and modes of vibration when subjected to vibration. These modal vibrations, coupled with increases in strain, lead to the excessive stretching of brain tissue, potentially causing MTBI. Modal analysis, identifying resonant frequencies, can facilitate the implementation of safety measures to mitigate or eliminate vibrations within these critical frequency ranges.

In a human brain, the pia mater, an extremely thin layer, serves as a barrier between the brain and the arachnoid and dura mater. The subarachnoid space, which is the area between them, is filled with cerebral spinal fluid (CSF), providing cushioning and nourishment to brain tissues [1,2]. Furthermore, the falx cerebri membrane, a deep fold of the dura mater, plays a crucial role in preventing the rotation of the brain inside the skull and separates the two hemispheres of the brain. The brain is categorized into the following three primary components: the cerebrum, cerebellum, and brain stem. The cerebrum, which is divided into two hemispheres, is connected by a bundle of fibers known as the corpus callosum [3]. Functioning as a bridge between the hemispheres, the corpus callosum also plays a vital role in the propagation of vibrations through the brain. The membrane-like behavior of the corpus callosum acts as a barrier, obstructing the propagation of vibrations and concentrating them near the corpus callosum, leading to increased strain in this area [4].

The Head Injury Criterion (HIC), established by Versace [5] in 1972, serves as a metric for assessing brain tolerance to head injuries. It quantifies the potential for injury based on the acceleration of the head’s center of mass (COM) following an impact. In crash test dummies, internal accelerometers gauge COM acceleration. While HIC cannot be considered a precise measure for brain injury criteria, it does indicate motion regions of the human head that may pose a risk to the brain. In numerical models and finite element methods (FEM), COM acceleration is computed during simulations and analyzed in post-processing. HIC is calculated using the following formula:

(1) $$\mathrm{HIC}=\max_{t_{1},t_{2}}\left\{{\left[\frac{1}{t_{2}-t_{1}}\int_{t_{1}}^{t_{2}}a(t)dt\right]}^{2.5}\right\}\left(t_{2}-t_{1}\right)$$

Here, *t*_1_ and *t*_2_ represent the initial and final times in seconds chosen to maximize HIC, and *a*(*t*) is the acceleration in g’s (9.81 m/s^2^) as a function of time. The time bounds are typically chosen for a maximum interval of 15 ms, known as HIC_15_, as suggested by Hodgson et al. [6] in 1972 for accurate concussion-level impact results. Mertz et al. [7] established injury risk curves using HIC_15_ and a Hybrid III crash test dummy. An HIC_15_ of 1000 corresponds to a 16% chance of skull fracture and a 17% chance of brain injury, according to their findings. However, HIC_15_ values for mild traumatic brain injury (MTBI) vary across the literature. Beckwith et al. [8] reported a mean HIC_15_ of 321.5 for concussive impacts in a study on football players. Other studies by entities such as the NFL show mean HIC_15_ values ranging from 249 to 557 [9,10,11]. Variance in values is influenced by sample size, age, and the diverse effects of different loading mechanisms and directions on brain acceleration. Notably, HIC_15_ has a limitation as it only considers linear acceleration, making it less accurate for rotationally dominated loadings.

Direct impact on the brain results from mechanical loads caused by assaulting the human head with a foreign object. The effects depend on impact magnitude, duration, location, and direction. Impacts are classified as perpendicular or oblique, with oblique impacts having both radial and tangential components, generating linear and rotational accelerations. Pure perpendicular impacts are rare, and statistics suggest that oblique impacts of 30–40° are more common in motorcycle accidents [11].

Biomechanics researchers have extensively employed FEM to investigate brain stresses and strains under various loading conditions. The application of FEM can be divided into structural-only models and fluid–structure interaction (FSI) models. For structural-only models, references include Zhang et al. [12], Doorly and Gilchrist [13], Kleiven [14], Miller et al. [15], Ghajari et al. [16], Yu et al. [17], Chaft et al. [18,19], and Rezaei et al. [20]. For FSI brain injury models, references include Zhu et al. [21,22], Luraghi [23], Terzano et al. [24], and Duckworth et al. [25,26]. In all cases, the biofidelity of these computational models requires an accurate geometric and mechanical description of brain elements, considering the complex nonlinear and time-dependent behavior of their materials. Notable studies by Chafi et al. [18,19] successfully implemented a Mooney–Rivlin hyper-viscoelastic model to monitor intracranial pressure (ICP) and brain shear stresses during dynamic and blast loading. Their work involved a comparison of stress and strain responses using both Ogden hyper-elastic and Mooney–Rivlin hyper-viscoelastic models for brains subjected to blast loading. Rezaei et al. [20] delved into the effects of a primary blast on ICP and shear stresses within the brain, considering blast scenarios in confined, semi-confined, and open spaces. Farid et al. [27] introduced a poro-hyper-viscoelastic, rate-dependent constitutive model for stress analysis in brain tissues. Ramzanpour et al. [28] applied an optimization FEM procedure for a visco-hyper-elastic characterization of human brain white matter. In a study by Kleiven et al. [14], FEM was employed to examine von Mises stresses in the skull and principal strains within the brain under the following two loading cases: purely perpendicular impact and an oblique impact at a 45° angle. Notably, their findings illustrated distinct outcomes for skull stress and brain strain between perpendicular and oblique impacts of the same magnitude. Perpendicular impact resulted in high skull stresses but relatively low principal strains in the brain. Conversely, oblique impact led to lower skull stresses but significantly higher brain strain. This relationship aligns with the work of Holbourn [29], who, using 2D gel models, asserted that rotational acceleration, not linear acceleration, is the primary cause of a majority of traumatic brain injuries (TBIs). Furthermore, McElhaney et al. [30] determined that the brain’s bulk modulus is 5–6 orders of magnitude larger than the shear modulus. Consequently, the brain exhibits greater sensitivity to rotational acceleration, deforming primarily in shear.

This research aims to use FEM and numerical techniques for modal analysis on the human brain model of structural-only type analysis to identify resonant frequencies. Utilizing LS-DYNA R9.3.0 finite element software [31], brain nodal displacement from a human head model will undergo DMD using MATLAB. DMD, which is capable of extracting dynamic characteristics without defining the underlying system, proves useful for both numerical and experimental data. This study seeks to identify a frequency range in which the brain is more susceptible to vibration, contributing to a better understanding of the brain in the frequency domain and the prevention of TBIs.

## 2. Modal Analysis and Dynamic Mode Decomposition

Modal analysis is the study of the dynamic properties of a system to determine the resonant frequencies, also called natural frequencies. When a structure experiences vibration at one of these resonant frequencies, it exhibits resonance. Resonance is caused by the interaction between the inertial and elastic properties of materials within a structure [32]. Resonance leads to excessive deformation and damage that compromises the functionality of the system. Regarding the brain, this excessive deformation correlates to an increase in strain and, subsequently, the stretching of axons and axonal injuries, which leads to concussions.

In mathematical terms, modal analysis is described by an eigenvalue problem. For direct methods of modal analysis, a non-damped linear system must be considered. In the equation of motion for a dynamical system, as follows: $[M]\{\ddot{u}\}+[K]\{u\}=0$, with $\left[M\right]$ as the equivalent mass matrix and $\left[K\right]$ as the equivalent stiffness matrix, $\left\{u\right\}$ is displacement, and $\left\{ü\right\}$ is acceleration. By assuming harmonic motion, a solution can be found using the following: $\left|[K]-[M]\omega^{2}\right|=0$. Solving this equation will result in n natural frequencies for an n-DOF system. The corresponding n mode shapes, $\phi_{i}$, can be determined using the following: $\phi_{i}=\left([K]-[M]\omega_{i}^{2}\right)A_{n}$, with $A_{n}$ as the normalized. Unfortunately, the brain cannot be analyzed using direct methods because it is a nonlinear material. Instead, numerical techniques must be implemented to approximate natural frequencies and the corresponding mode shapes.

DMD was first formulized by Schmid [33], who was seeking to develop a method of modal analysis that was equally applicable for both experimental and numerical data. Schmid aimed to create a “matrix-free” formulation that relied on gathered data and did not depend on any information regarding the underlying system matrix. Schmid concentrated on a data-based approach rather than a model-based approach. DMD is unique and quite similar to other numerical techniques, such as proper orthogonal decomposition (POD) and bi-orthogonal decomposition (BOD). POD employs energy ranking to compare orthogonal structures gathered from snapshots of flow vectors. The POD modes are gathered from singular value decomposition as follows: $V_{1}^{N-1}=U\Sigma W^{H}$, where $V_{1}^{N-1}$ is the snapshot matrix, U contains the spatial modes, W contains the temporal modes, and the diagonal values of Σ represent the energy ranks. POD has been successfully used for fluid flow by Berkooz et al. [34], but it has two main limitations. First, sometimes energy is not the proper parameter to rank modes, and secondly, POD uses second-order statistics, so phase information is lost. BOD is very similar to POD and determines the eigenvectors for both the spatial and temporal matrices. Yet again, BOD uses second-order statistics and loses valuable transient information. DMD does not lose this phase information and is superior to POD in highly transient events, such as head impact. DMD is better equipped to capture the intrinsic nonlinearities present in the human brain [34].

To understand DMD, first consider brain nodal displacement collected as a series of snapshots in time. Each snapshot is separated by a constant time interval ∆t. This sequence of N snapshots is collected as a series of column vectors given by the matrix $V_{1}^{N}$, as follows:

(2) $$V_{1}^{N}=\{v_{1},v_{2},v_{3},\ldots ,v_{N-1},v_{N}\},$$

where, $v_{i}$ represents the ith snapshot of nodal displacement data. $V_{1}^{N}$ is an *M × N* matrix consisting of *M* nodal displacements separated into *N* snapshots in time. Furthermore, this study assumes the presence of a linear mapping coefficient *A* that connects the flow field $v_{i}$ to the subsequent flow field ${\;v}_{i+1}$, such that $v_{i+1}=Av_{i}$, and *A* is assumed to remain constant throughout the sequence. In the case of a nonlinear system such as the brain, this amounts to a linear tangent approximation between snapshots. Thus, in combination we have the following:

(3) $$V_{1}^{N}=\{v_{1},Av_{1},A^{2}v_{1},\ldots ,A^{N-2}v_{1},A^{N-1}v_{1}\}.$$

The goal of DMD is then to extract the dynamical characteristics of *A* based on the data described by $V_{1}^{N}.$ In a complex dynamical system like the human brain with thousands or millions of nodes, constructing matrix *A* can be computationally expensive and inefficient. Therefore, to avoid constructing matrix *A*, this study utilizes singular value decomposition to construct a much smaller matrix $\tilde{S}$. The matrix $\tilde{S}$ is related to *A* through a similarity transform; thus, the dynamic characteristics of $\tilde{S}$ are the same as *A*. To construct $\tilde{S}$, first, separate the snapshot matrix $V_{1}^{N}$ into two submatrices $V_{1}^{N-1}$ and $V_{2}^{N}$ as follows:

(4) $$\begin{array}{l} V_{1}^{N-1}=\left\{v_{1},v_{2},v_{3},\ldots ,v_{N-2},v_{N-1}\right\},\\ V_{2}^{N}=\left\{v_{2},v_{3},v_{4},\ldots ,v_{N-1},v_{N}\right\},\\ V_{2}^{N}=AV_{1}^{N-1} \end{array}$$

Using singular value decomposition (SVD), $V_{1}^{N-1}$ is decomposed into matrices U,Σ, and W using the following: $V_{1}^{N-1}=U\Sigma W^{H}.$ By inserting $U\Sigma W^{H}$ into Equations (3) and reorganizing the matrices, $\tilde{S}$ can be determined using the following:

(5) $$U^{H}AU=U^{H}V_{2}^{N}W\Sigma^{-1}\equiv \tilde{S}$$

As discussed before, $\tilde{S}$ is related to A through a similarity transformation. Looking back at POD, the matrix *U* contains the spatial modes of the system, and from Equation (2), it is known that matrix *A* represents a linear mapping coefficient one step forward in time. So, it can be seen from Equation (4) that $\tilde{S}$ better describes the transient properties of the system. Additionally, the eigenvalues of $\tilde{S}$ are the eigenvalues of *A*, and the eigenvectors of A can be determined from the eigenvectors of $\tilde{S}$ as follows: $\phi_{i}=Uy_{i}$, where $y_{i}$ is the ith eigenvector of $\tilde{S}$, and $\phi_{i}$ is the ith eigenvector of *A*. The future state of each eigenvector, or mode shape, can be predicted for all time in the future by using the Koopman operator [35].

## 3. Dynamic Mode Decomposition of the Human Brain Model

**Figure 1:**
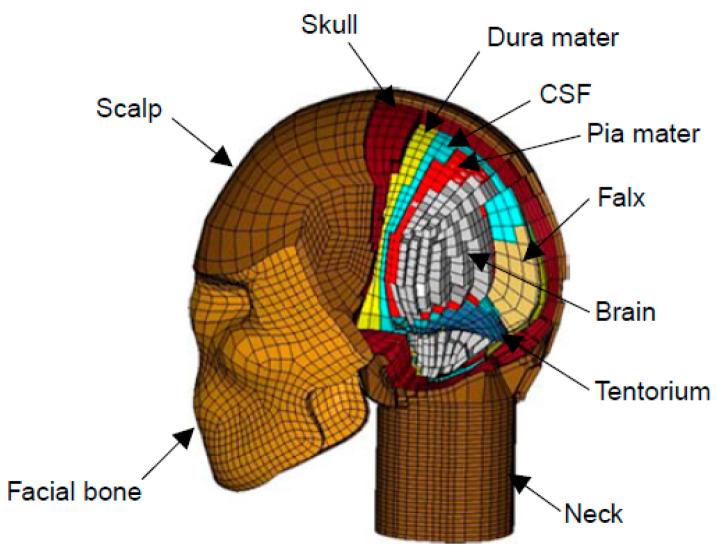
Identification of the main components of the human head [1].

The three-dimensional finite element head model employed in this study was developed within our research group, as detailed in Chafi et al. [18,19] and Rezaei et al. [20]. The geometry of the model was derived from computed tomography (CT) scans acquired from the National Institute of Health (NIH). The model underwent validation through the simulation of impact tests conducted by Nahum et al. [36]. The validation process involved a direct comparison of predicted increased intracranial pressure (ICP) time histories against experimentally obtained values. The head–neck model shown in Figure 1 comprehensively models major components of the human head using 23,361 eight-nodded brick elements and 5344 four-nodded shell elements. Various components of the head, such as the facial bone, scalp, skull, brain, neck, and cerebrospinal fluid (CSF), were modeled using eight-nodded brick elements. Membranes like the dura mater, pia mater, falx, and tentorium were represented using four-nodded shell elements. Linear elastic materials were employed to model the scalp, skull, neck, dura mater, pia mater, and tentorium. The CSF, characterized as an elastic fluid, was modeled to avoid shear stress but allow for the transmission of hydrostatic stress. The brain material was modeled using a Mooney–Rivlin hyper-viscoelastic model (Mendis et al. [37]).

**Figure 2:**
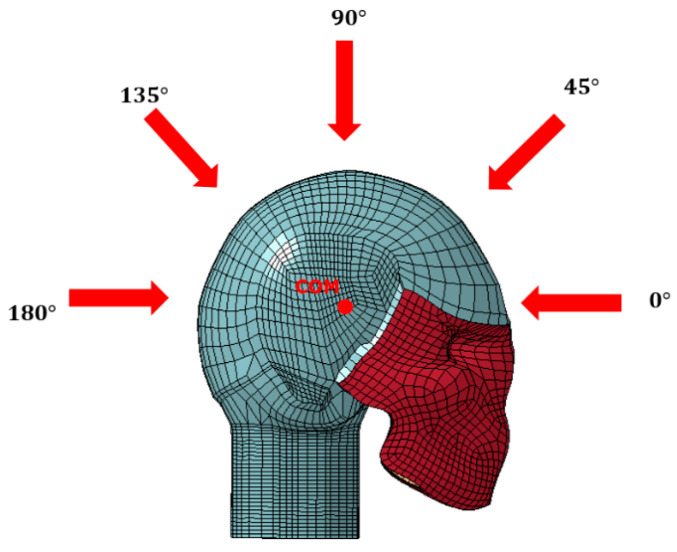
Depiction of the varying angles of impact within the sagittal plane and the location of the COM of the head [1].

As shown in Figure 2, the primary focus of this study is to investigate how varying the direction of impact influences dynamic modes within the skull. Impact simulations were conducted in 45° increments within the sagittal plane, ranging from 0° to 180°, with the center of mass (COM) of the head serving as the center of rotation. The anterior direction was designated as 0°.

**Figure 3:**
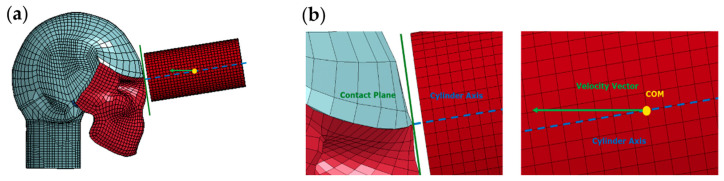
(**a**) Schematic depicting the overall setup of direct impact using a steel cylinder. (**b**) Detailed schematic of direct impact setup at the contact plane (**left**) and the COM of the steel cylinder (**right**). It is important to note that the cylinder axis is normal to the contact plane and the velocity vector is not collinear with the cylinder axis [1].

Direct impacts were generated using a 5.58 kg steel cylinder, positioned so that its COM coincided with the designated impact angle. Recognizing that the human head is not a perfect sphere and to ensure flush contact, the cylinder was slightly rotated about its COM, aligning the axial axis of the cylinder at a 90° angle with the tangential direction of the contact plane. The cylinder was then given an initial velocity in the direction of the impact angle. As a result, the velocity vector and cylinder axis were not collinear, and the velocity vector was not normal to the contact plane. Detailed illustrations of these directions and adjustments for the 0° impact angle are provided in Figure 3.

The initial velocities were adjusted based on the desired HIC_15_ values, aiming for a range of 350–450 to ensure concussive level impacts. The mean HIC_15_ for concussive impacts, as determined by Beckwith et al. [7], was 321.5. Using educated trial and error, the initial velocities for each impact angle were fine-tuned until the calculated HIC_15_ fell within the specified range. The corresponding initial velocities and HIC_15_ values for each impact angle are presented in Table 1.

To isolate brain displacement due to vibration, rigid body motion must be subtracted from the system. This subtraction was accomplished using a follow command within LS-DYNA. A follow plane, which was constructed by selecting three nodes of the skull, established a new coordinate system that tracked the rigid body motion of the skull. This eliminated the rigid body motion of the brain in all six Cartesian degrees of freedom. The resulting nodal brain displacements, described in this new coordinate system, were solely attributed to vibration.

**Figure 4:**
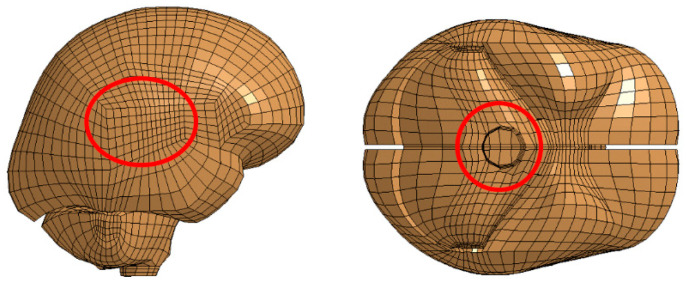
Region of nodes in the cerebrum near the corpus callosum (**left**). Region of nodes in the cerebellum near the brain stem (**right**) [1].

For each impact direction, the following two groups of nodes were chosen: one in the cerebrum near the corpus callosum and the other in the occipital lobe near the brain stem. These selections were based on previous studies indicating that shear waves and resulting strains concentrate near the corpus callosum and brain stem [4,38,39]. The locations of these node selections are illustrated in Figure 4.

To extract the dynamic modes of vibration, the selected nodes were entered into the prepared DMD code developed for this study. The functionality and accuracy of the DMD code were verified by comparing the generated solutions with a sinusoidal waveform previously studied by Laksari et al. [39]. The sinusoidal signal, *u*(*t*), took the following form:

(6) $$u(t)=\sin\left(k_{1}x+\omega_{1}t\right)e^{-\lambda_{1}t}+\sin\left(k_{2}x+\omega_{2}t\right)e^{-\lambda_{2}t}$$

where $k_{1}=10,k_{2}=30,\omega_{1}=20,\omega_{2}=60,\lambda_{1}=-3,$ and $\lambda_{2}=-5$. This signal is a combination of two distinct linear spatiotemporal substructures that are decaying over time. As discussed earlier, data can be truncated for the DMD method to reduce computational cost. The singular matrix Σ provides scaling for the modal amplitudes of dynamic modes. This matrix is determined during SVD. The diagonal values of Σ show that modal amplitudes become negligible after five modes. Thus, the three matrices determined using SVD are truncated into three 5×5 matrices, greatly reducing the overall computational cost.

**Figure 5:**
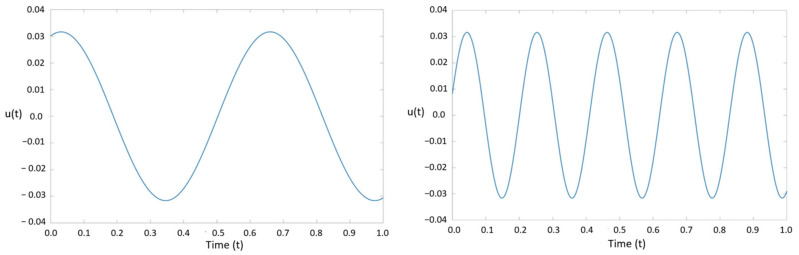
The first mode of vibration at a frequency of 3 (**left**). The third mode of vibration at a frequency of 5 (**right**).

Using DMD, the first and third modes extracted from the system were plotted in Figure 5. Then, the frequencies and decay rates of each mode were determined using the Koopmans operator [26]. The Koopmans operator is a complex number in which the real part represents the mode frequency, and the imaginary part represents the decay rate. The first mode had a frequency of three and a decay rate of 19.9916, and the third mode had a frequency of five with a decay rate of 59.9573. It is important to note that the second mode was skipped because it represents the complex conjugate of the first mode. The second mode has the same decay rate as the first mode, but its frequency is negative.

**Figure 6:**
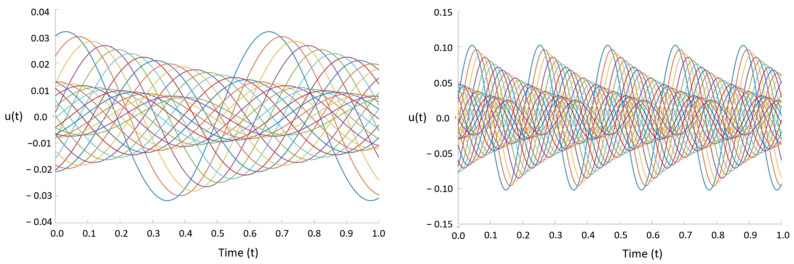
The first mode decaying at a rate of 19.9916 (**left**). The third mode decaying at a rate of 59.9573 (**right**).

Furthermore, using Equation (5), the future states of the first and third modes were predicted as they decay over time (Figure 6). The frequencies, decay rates, and future state predictions determined using this DMD code are identical to those determined by Laksari et al. [28]. This verified the functionality of the novel DMD code and its ability to be applied to a modal analysis of the human brain.

## 4. Numerical Solutions and Discussion

**Figure 7:**
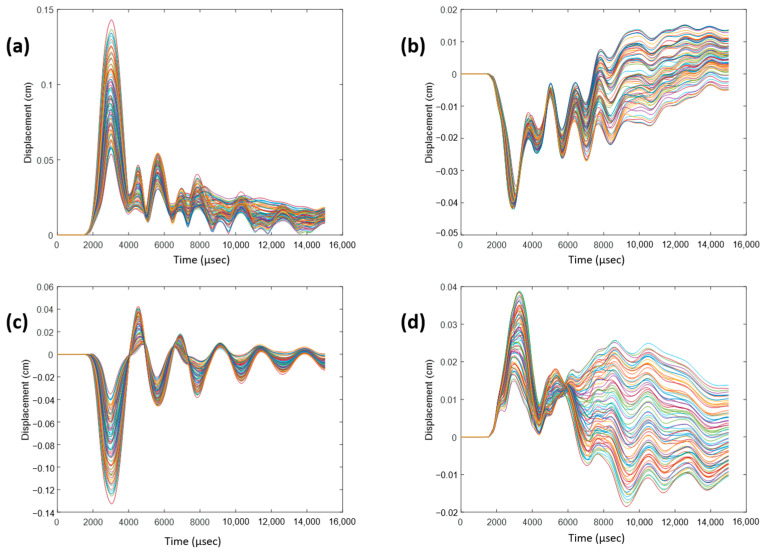
(**a**) Total relative displacement of nodes near the corpus callosum for 0° impact angle. (**b**) Relative displacement for 0° impact angle in coronal direction. (**c**) Relative displacement for 0° impact angle in the sagittal direction. (**d**) Relative displacement for 0° impact angle in the axial direction. The lines colored show the different values at the different nodes selected.

**Figure 8:**
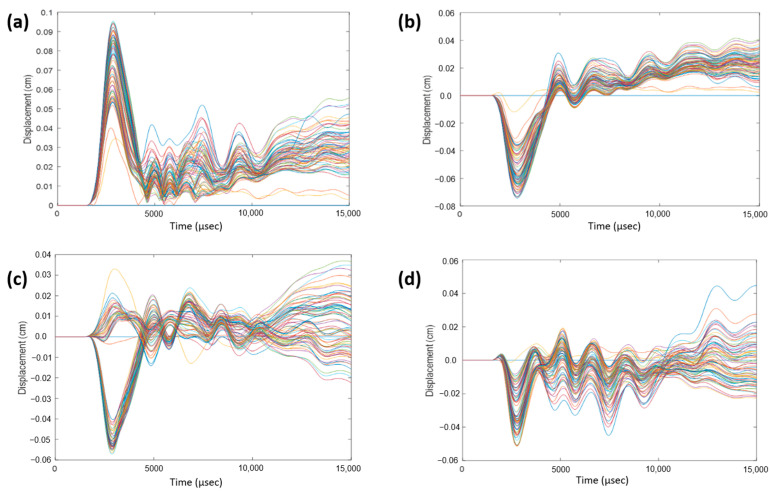
(**a**) Total relative displacement of nodes near the brain stem for 180° impact angle. (**b**) Relative displacement for 180° impact angle in coronal direction. (**c**) Relative displacement for 180° impact angle in the sagittal direction. (**d**) Relative displacement for 180° impact angle in the axial direction. The lines colored show the different values at the different nodes selected.

To monitor displacement, a 3D coordinate system was established with the following three axes: coronal, sagittal, and axial, defined as the normal directions to the coronal, sagittal, and axial planes, respectively. Within the cerebrum, near the corpus callosum, a selection of nodes was made. For each impact direction, the total relative displacements, as well as displacements in the sagittal, coronal, and axial directions, were monitored over a 15-ms duration and plotted. Figure 7 illustrates displacements for a 0° impact angle, serving as an example. This process was iterated for a group of nodes within the cerebellum near the brain stem, and Figure 8 exhibits displacements for a 180° impact angle near the brain stem. Observations reveal sinusoidal behavior for each impact angle, albeit with variations across impact angles and displacement directions. Despite subtracting the rigid body motion of the skull from total displacement, remnants of such motion persist in the coronal and axial directions. The sagittal direction exhibits the most consistent sinusoidal behavior due to minimal rigid body motion, with a majority of the displacement attributable to vibration. Notably, nodes near the brain stem exhibit suboptimal sinusoidal behavior due to a rigid support boundary condition applied to the bottom surface of the neck. This condition restricts spine movement in the inferior axial direction, causing the spine to exert force on the brain stem in the superior axial direction. Consequently, additional rigid body motion is introduced, which is not fully accounted for by the motion of the skull.

### 4.1. Data Truncation

Subsequently, the truncation point of the system was determined by examining the diagonal values of the singular matrix obtained through SVD. The trace of this singular matrix serves as an indicator of the total variance within the mathematical system, equating mathematical variance to the physical energy of the system. Dividing each diagonal element of the singular matrix by its trace enables the determination of the percentage of energy contribution for each mode. Using the total displacements of nodes near the corpus callosum and brain stem, variances for each impact angle were computed. The results revealed a dominant mode for each impact angle, contributing approximately 70–80% of the system’s variance. Moreover, for each impact angle, nearly all the energy of the system was consumed within the first six modes. Given that the modes are complex conjugate pairs, this truncation to six modes equates to three physical modes, as discussed earlier. This strategic truncation not only significantly reduces computational costs but also ensures an accurate representation of the system.

### 4.2. Modal Frequencies

**Figure 9:**
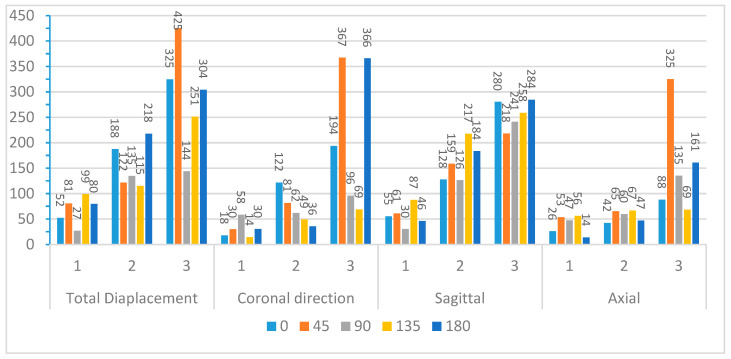
Modal frequencies (Hz) for displacement of nodes near the corpus callosum.

**Figure 10:**
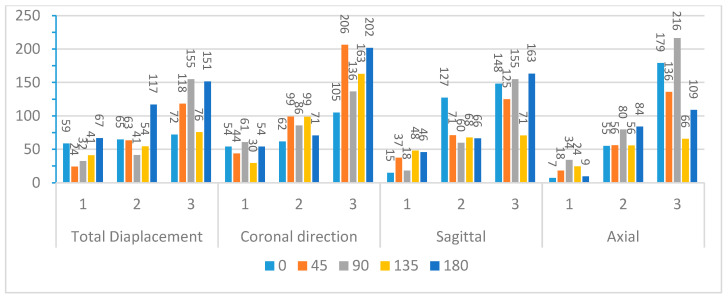
Modal frequencies (Hz) for displacement of nodes near the brain stem.

After truncating the system, modal frequencies, modal amplitudes, and decay rates were determined for each impact angle. Figure 9 and Figure 10 compare the first three modal frequencies for nodes near the corpus callosum and the brain stem, respectively. The modes are ordered from the lowest to the highest modal frequency (Mode 1 to Mode 3). These frequencies are compared for total displacement and displacement in the sagittal, coronal, and axial directions at each of the five impact angles. Observations from the graphs indicate that while modal frequencies vary across impact angles for each location and displacement direction, there is a consistent presence of three distinct modal frequencies. Moreover, modal frequencies seem to exhibit some level of mirroring over the coronal plane. Specifically, the 45° impact angle shows similarities with the 135° impact angle, and the 0° impact angle aligns with the 180° impact angle. Although not identical, this pattern is expected due to the geometric and material differences at the impact location, as well as the influence of the neck boundary condition. Analyzing nodes near the corpus callosum, modal frequencies in the sagittal direction display the most distinction among the three modal frequencies. This aligns with the earlier observation that displacement in the sagittal direction exhibits better sinusoidal behavior, suggesting DMD’s enhanced accuracy in extracting dynamic modes of vibration. Displacement in the coronal direction further supports this claim, as Mode 3 frequencies for the 0° and 180° impact angles show abnormally high modal frequencies, indicating increased rigid body motion that may reduce DMD accuracy.

**Figure 11:**
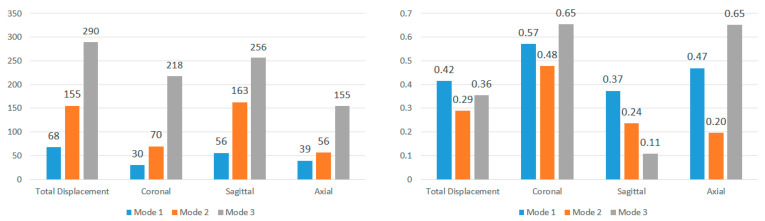
Average modal frequencies (Hz) across impact angles (**left**) and their respective coefficients of variance (**right**) for nodes near the corpus callosum.

**Figure 12:**
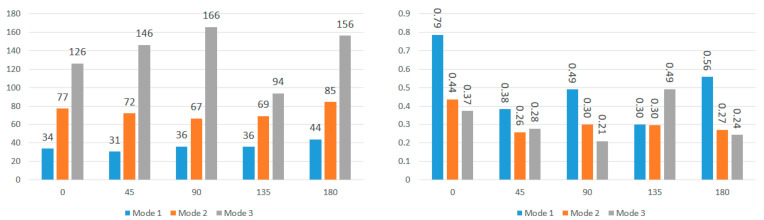
Average modal frequencies (Hz) across impact angles (**left**) and their respective coefficients of variance (**right**) for nodes near the brain stem.

To further explore these patterns, Figure 11 and Figure 12 present the average modal frequencies across the five impact angles for each displacement direction, offering a clearer picture. Additionally, the coefficient of variance (CV), calculated as the standard deviation divided by the average modal frequency, is provided for each mode and displacement direction. CV values highlight the variance between different impact angles, with higher values indicating a significant impact on modal frequencies. Typically, a CV upper limit of 0.30 is considered high, and the high CV values here emphasize the substantial effect of impact angles on modal frequencies, complicating engineering considerations for safety designs. Examining CV values for nodes near the corpus callosum, the sagittal direction consistently exhibits the lowest CV across all three modes, particularly in Mode 3. This statistical support aligns with the visual observations from the bar graphs. Although CV values are generally high, exceeding the typical threshold, they underscore the impact angle’s substantial effect on modal frequencies during brain impact events. This complexity implies the need for the separate consideration of various impact angles in safety designs, covering a broad frequency range.

**Figure 13:**
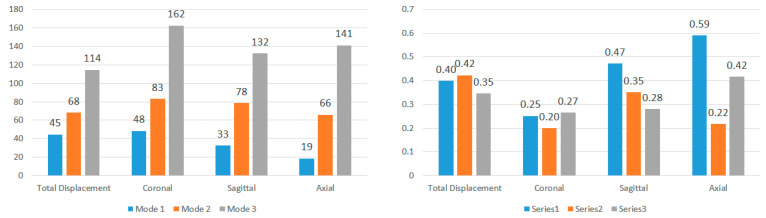
Average modal frequencies (Hz) across displacement directions (**left**) and their respective coefficients of variance (**right**) for nodes near the corpus callosum.

**Figure 14:**
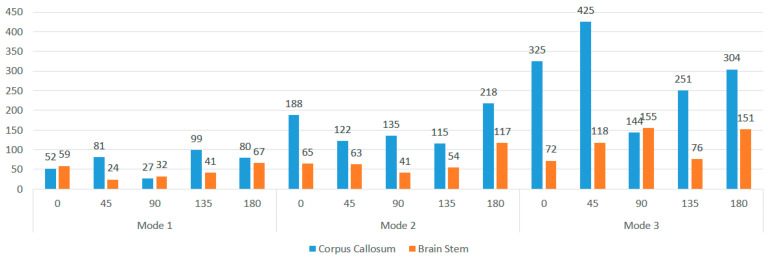
Average modal frequencies (Hz) across displacement directions (**left**) and their respective coefficients of variance (**right**) for nodes near the brain stem.

**Figure 15:**
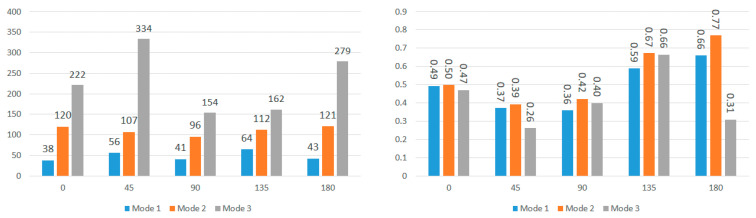
Comparison of modal frequencies (Hz) for each impact angle using total displacement of nodes near the corpus callosum and brain stem (Modes 1, 2, and 3).

Further analysis explores the effects of displacement direction by averaging modal frequencies across displacement directions for each impact angle (Figure 13 and Figure 14). Once again, CV values surpass the 0.30 threshold for nearly all impact angles in both locations, indicating that the choice of displacement direction has a notable impact on determined modal frequencies. The higher CV values for modal frequencies averaged across displacement directions compared to those averaged across impact angles highlight the increased influence of displacement direction on modal frequencies. To understand regional effects, modal frequencies were compared between nodes near the corpus callosum and the brain stem for each impact angle, eliminating the impact of angles and displacement directions. This direct comparison, illustrated in Figure 15 for total displacement, indicates significant differences in modal frequencies between these two regions. With exceptions for two impact angles in Mode 1 and one impact angle in Mode 3, corpus callosum modal frequencies are consistently higher than those near the brain stem. A possible explanation involves the interaction between the brain stem and spine, influencing displacement and potentially shifting modal frequencies. This comparative data emphasizes the considerable impact of location on the modal frequencies experienced during an impact event.

### 4.3. Modal Amplitudes and Decay Rates

In addition to frequencies, this study determined the modal amplitudes and decay rates for each impact angle, location, and mode. DMD provides modal amplitudes for each node, and there is no consensus on the correct method to compare modes in the literature. Two common approaches are to average the modal amplitudes for each node or use the L2-norm. This study opted for the L2-norm, normalizing the magnitude of Modes 1, 2, and 3 for each loading case and displacement direction by dividing the L2-norm of each mode by the maximum L2-norm value. The decay rates were determined using the imaginary part of the relevant equation.

Table 2, Table 3, Table 4, Table 5 and Table 6 present the normalized modal amplitudes and decay rates for the total displacement of nodes near the corpus callosum for each impact angle. However, the DMD code did not distinctly identify the dominant modes of vibrations for different impact angles. Normalized modal amplitudes close to one imply that, comparatively, the modes contribute equally to the total displacement and the vibrational characteristics of the brain. This challenges the identification of a sensitive frequency range for the brain. While three distinct modes of vibration are evident, distinguishing the dominant mode (Mode 1, 2, or 3) becomes challenging. This complexity hampers the design of helmets or safety devices to protect against resonance, as it is unclear which frequency range to suppress

Moreover, the decay rates determined using the DMD code appear very high, and in some cases, were zero. High decay rates suggest very high damping and a short duration of vibration, contrary to the widespread belief in the literature that brain vibration lasts for a significant duration, often exceeding 100 ms. A decay rate of zero implies a quasi-static wave that does not decrease in amplitude, which is an impossibility in physical terms. This suggests potential limitations or inaccuracies in the obtained solutions.

While the DMD code successfully determined numerical values for mode shapes and their decay over time, technological limitations prevented the application of these values to form 3D mode shapes. The modal displacements for each direction need to be reintegrated into the finite element analysis model with the correct node and spatial arrangement for the mode shapes to have physical meaning and correlation to the brain. As it stands, the mode shapes represent a collection of random displacements without a clear physical interpretation.

Certainly, the accuracy of dynamic mode decomposition (DMD) hinges on various computational parameters. The proximity of the derived frequencies to the exact injury regions relies on the precision of the modeling process. Factors such as finite element formulations, boundary conditions, brain material modeling, and the arrangement of head elements significantly influence the fidelity of the results.

## 5. Conclusions

A successful DMD code in conjunction with the data from finite element analysis of the human head was developed, showcasing its capabilities and applications. Through the use of SVD and DMD following the data from finite element analysis of a human head, this study demonstrated that brain vibration can be effectively described using three or fewer modes of brain vibration. Three modal frequencies were identified with frequency ranges of (44–68) Hz, (68–155) Hz, and (114–299) Hz. In different loading scenarios to the human head, nearly all the system’s variance was captured within the first three modes. For each case, the DMD code successfully extracted modal amplitudes, decay rates, and numerical mode shapes. The investigation also sheds light on the impact of various factors, such as impact angle, displacement direction, and brain regions. The data revealed significant influences of these variables on modal frequencies and decay rates. Impact angle, in particular, led to distinct modal frequencies, with some evidence of mirroring over the coronal plane for specific modes and displacement angles. Different displacement directions exhibited varied modal frequencies, emphasizing the importance of considering all three principal displacement directions. This study also highlighted notable differences in modal frequencies based on whether finite element nodes were located near the corpus callosum or the brain stem. Frequencies were lower for nodes near the brain stem, and this study attributes this to the impact of boundary conditions and the influence of the spine pressing on the brain stem.

While the DMD procedure proved to be a potent and effective numerical technique, this study acknowledged its dependency on the quality and quantity of the finite element data. There is recognized room for improvement in the methodology and data collection, presenting an opportunity to enhance results and gain deeper insights. Despite being an initial step, this study underscores the exciting and promising future of DMD in advancing our understanding of traumatic brain injury (TBI) and the complexities of the human brain.

## Figures and Tables

**Table 1:** Initial velocities and $HIC_{15}$ for different impact angles.

Impact Angle	0°	45°	90°	135°	180°	Average
Initial Velocity (m/s)	5.00	4.53	3.60	4.53	5.00	4.53
HIC_15_	395.35	398.35	355.41	398.83	407.22	391.03

**Table 2:** Modal frequency, amplitude, and decay rates for nodes near the corpus callosum and a 0° impact angle. Modal amplitudes were normalized using the maximum modal amplitude to provide a quality comparison.

Modal Frequency (Hz)	Normalized Modal Amplitude	Decay Rate (Hz)
52.242	1.000	94.535
187.641	0.985	1334.842
324.639	0.811	697.619

**Table 3:** Modal frequency, amplitude, and decay rates for nodes near the corpus callosum and a 45° impact angle. Modal amplitudes were normalized using the maximum modal amplitude to provide a quality comparison.

Modal Frequency (Hz)	Normalized Modal Amplitude	Decay Rate (Hz)
80.789	0.989	337.736
121.832	1.000	0.000
425.477	0.953	3026.153

**Table 4:** Modal frequency, amplitude, and decay rates for nodes near the corpus callosum and a 90° impact angle. Modal amplitudes were normalized using the maximum modal amplitude to provide a quality comparison.

Modal Frequency (Hz)	Normalized Modal Amplitude	Decay Rate (Hz)
27.122	1.000	84.112
134.723	0.993	1400.362
144.055	0.971	483.238

**Table 5:** Modal frequency, amplitude, and decay rates for nodes near the corpus callosum and a 135° impact angle. Modal amplitudes were normalized using the maximum modal amplitude to provide a quality comparison.

Modal Frequency (Hz)	Normalized Modal Amplitude	Decay Rate (Hz)
98.862	1.000	0.000
114.965	0.982	713.222
251.098	0.974	1571.536

**Table 6:** Modal frequency, amplitude, and decay rates for nodes near the corpus callosum and a 180° impact angle. Modal amplitudes were normalized using the maximum modal amplitude to provide a quality comparison.

Modal Frequency (Hz)	Normalized Modal Amplitude	Decay Rate (Hz)
79.785	1.000	0.000
217.687	0.983	756.033
304.113	0.977	774.457

## Data Availability

Detailed information can be found at https://hdl.handle.net/10365/31804 (accessed on 7 September 2023).

## References

[1] McLean J. (2020). Modal Analysis of the Human Brain Using Dynamic Mode Decomposition. Master’s Thesis.

[2] Tse K.M., Lim S.P., Tan V.B.C., Lee H.P. (2014). A review of head injury and finite element head models. Am. J. Eng. Technol. Soc..

[3] Rhoton A. (2002). The cerebrum. Neurosurgery.

[4] Gurdjian E., Hodgson V., Thomas L., Patrick L., Neurosurg L. (1968). Significance of relative movements of scalp, skull, and intracranial contents during impact injury of the head. J. Neurosurg..

[5] Versace J. A review of the severity index. Proceedings of the 15th Stapp Car Crash Conference ASE.

[6] Hodgson V.R., Thomas L.M. (1972). Effect of Long-Duration Impact on Head. SAE Technical Paper 72095.

[7] Mertz H.J., Prasad P., Irwin A.L. (1997). Injury Risk Curves for Children and Adults in Frontal and Rear Collisions. SAE Tech. Pap. Ser..

[8] Beckwith J.G., Greenwald R.M., Chu J.J., Crisco J.J., Rowson S., Duma S.M., Broglio S.P., Mcallister T.W., Guskiewicz K.M., Mihalik J.P. (2013). Head Impact Exposure Sustained by Football Players on Days of Diagnosed Concussion. Med. Sci. Sports Exerc..

[9] Gordon A.G. (2004). Concussion in Professional Football: Reconstruction of Game Impacts and Injuries. Neurosurgery.

[10] Funk J.R., Duma S.M., Manoogian S.J., Rowson S. (2007). Biomechanical risk estimates for mild traumatic brain injury. Annu. Proc. Assoc. Adv. Automot. Med..

[11] Chang L., Guo Y., Huang X., Xia Y., Cai Z. (2021). Experimental study on the protective performance of bulletproof plate and padding materials under ballistic impact. Mater. Des..

[12] Zhang L., Yang K.H., King A.I. (2004). A Proposed Injury Threshold for Mild Traumatic Brain Injury. J. Biomech. Eng..

[13] Doorly M.C., Gilchrist M.D. (2006). The use of accident reconstruction for the analysis of traumatic brain injury due to head impacts arising from falls. Comput. Methods Biomech. Biomed. Eng..

[14] Kleiven S. (2007). Predictors for Traumatic Brain Injuries Evaluated through Accident Reconstructions. SAE Technical Paper 2007-22-0003.

[15] Miller L.E., Urban J.E., Stitzel J.D. (2007). Validation Performance Comparison for Finite Element Models of the Human Brain. Comput. Methods Biomech. Biomed. Eng..

[16] Ghajari M., Hellyer P.J., Sharp D.J. (2017). Computational Modelling of Traumatic Brain Injury Predicts the Location of Chronic Traumatic Encephalopathy Pathology. Brain.

[17] Yu X., Azor A., JSharp D., Ghajari M. (2020). Mechanisms of Tensile Failure of Cerebrospinal Fluid in Blast Traumatic Brain Injury. Extrem. Mech. Lett..

[18] Chafi M.S., Karami G., Ziejewski M. (2010). Biomechanical Assessment of Brain Dynamic Responses Due to Blast Pressure Waves. Ann. Biomed. Eng..

[19] Chafi M.S., Karami G., Ziejewski M. (2009). Numerical analysis of blast-induced wave propagation using FSI and ALE multi-material formulations. Int. J. Impact Eng..

[20] Rezaei A., Salimi Jazi M., Karami G. (2014). Computational Modeling of Human Head under Blast in Confined and Open Spaces—Primary Blast Injury. Int. J. Numer. Methods Biomed. Eng..

[21] Zhou Z., Li X., Kleiven S. (2019). Fluid–Structure Interaction Simulation of the Brain–Skull Interface for Acute Subdural Haematoma Prediction. Biomech. Model. Mechanobiol..

[22] Zhou Z., Zhou Z., Li X., Li X., Kleiven S., Kleiven S. (2019). Biomechanics of Acute Subdural Hematoma in the Elderly: A Fluid-Structure Interaction Study. J. Neurotrauma.

[23] Luraghi G., Wu W., De Gaetano F., Matas J.F.R., Moggridge G.D., Serrani M., Stasiak J., Costantino M.L., Migliavacca F. (2017). Evaluation of an aortic valve prosthesis: Fluid-structure interaction or structural simulation?. J. Biomech..

[24] Terzano M., Spagnoli A., Dini D., Forte A.E. (2022). Fluid-solid interaction in the rate-dependent failure of brain tissue and biomimicking gels. Eng. Struct..

[25] Duckworth HAzor A., Wischmann N., Zimmerman K.A., Tanini I., Sharp D.J., Ghajari M. (2022). A Finite Element Model of Cerebral Vascular Injury for Predicting Microbleeds Location. Front. Bioeng. Biotechnol..

[26] Duckworth H., Sharp D.J., Ghajari M. (2021). Smoothed Particle Hydrodynamic Modelling of the Cerebrospinal Fluid for Brain Biomechanics: Accuracy and Stability. Int. J. Numer. Methods Biomed. Eng..

[27] Farid M.H., Ramzanpour M.R., McLean J., Ziejewski M., Karami G. (2019). A poro-hyper-viscoelastic rate-dependent constitutive modeling for the analysis of brain tissues. J. Mech. Behav. Biomed. Mater..

[28] Ramzanpour M.R., Hosseini-Farid M., McLean J., Ziejewski M., Karami G. (2020). Visco-hyperelastic characterization of human brain white matter micro-level constituents in different strain rates. Med. Biol. Eng. Comput..

[29] Holbourn A. (1943). Mechanics of Head Injuries. Lancet.

[30] Mcelhaney J. (1982). Biomechanics. Mechanical Properties of Living Tissues by Y. C. Fung. Med. Phys..

[31] (2006). LS-DYNA Finite Element Software.

[32] Schwarz B.J., Richardson M.H. Experimental Modal Analysis. Proceedings of the CSI Reliability Week.

[33] Schmid P.J. (2010). Dynamic mode decomposition of numerical and experimental data. J. Fluid Mech..

[34] Berkooz G., Holmes P., Lumley J.L. (1993). The proper orthogonal decomposition in the analysis of turbulent flows. Annu. Rev. Fluid Mech..

[35] Mann J., Kutz J.N. (2016). Dynamic mode decomposition for financial trading strategies. Quant. Financ..

[36] Nahum A.M., Smith R., Ward C.C. (1977). Intracranial Pressure Dynamics during Head Impact. SAE Technical Paper 770922. https://saemobilus.sae.org/papers/intracranial-pressure-dynamics-head-impact-770922.

[37] Mendis K.K., Stalnaker R.L., Advani S.H. (1995). A constitutive relationship for large deformation finite element modeling of brain tissue. J. Biomech. Eng..

[38] Zhang L., Yang K.H., King A.I. (2001). Comparison of brain responses between frontal and lateral impacts by finite element modeling. J. Neurotrauma.

[39] Laksari K., Kurt M., Babaee H., Kleiven S., Camarillo D. (2018). Mechanistic insights into human brain impact dynamics through modal analysis. Phys. Rev. Lett..

